# Operative Management of Hip Fractures Within 24 Hours in the Elderly is Achievable and Associated With Reduced Opiate Use

**DOI:** 10.1177/21514593221116331

**Published:** 2022-08-17

**Authors:** Sachin Allahabadi, Mohammad Roostan, Erika Roddy, Derek T. Ward, Stephanie Rogers, Candace Kim

**Affiliations:** 1 Department of Orthopaedic Surgery, 8785 University of California San Francisco, San Francisco, CA, USA; 2 Department of Internal Medicine, Division of Geriatrics, 8785 University of California San Francisco, San Francisco, CA, USA

**Keywords:** geriatric trauma, hospitalist, systems of care, fragility fractures, geriatric medicine, pain

## Abstract

**Introduction:**

Morbidity and mortality benefits have been associated with prompt surgical treatment of geriatric hip fractures. The purpose of this study was to evaluate the impact of early (≤24 hr) vs delayed (>24 hr) time to operating room (TTOR) on 1) hospital length of stay and 2) total and post-operative opiate use in geriatric hip fractures.

**Materials and Methods:**

This study was a retrospective review of patients ≥65 years-old at the time of admission for surgery for hip fracture at a Level II academic trauma center. Outcome measures were length of stay (LOS), oral morphine equivalents (OME) throughout hospitalization. Patients were stratified into early and delayed TTOR groups and comparisons were made between groups.

**Results:**

Between the early (n = 75, 80.6%) and late (n = 18, 19.4%) groups, there were no differences in age, fracture pattern, type of treatment, preoperative opiate use, and perioperative non-oral pain management. The early group trended toward shorter total LOS (108.0 ± 67.2 hours vs 144.8 ± 103.7 hours, *P* = .066), but not post-operative LOS. Total OME usage was less in the early intervention group (92.5 ± 188.0 vs 230.2 ± 296.7, *P* = .015), in addition to reduced post-operative OME (81.3 ± 174.9 vs 213.3 ± 271.3, *P* = .012). There were no differences in evaluated potential delay sources such as primary language, use of surrogate decision makers, or need for advanced imaging.

**Discussion:**

Surgical treatment of geriatric hip/femur fractures in ≤24 hours from presentation is achievable and may be associated with reduced total inpatient opiate use, although daily use did not differ.

**Conclusion:**

Establishing institutional TTOR goals as part of an interdisciplinary hip fracture co-management clinical pathway can facilitate prompt care and contribute to recovery and less opiate use in these patients with highly morbid injuries.

## Introduction

Hip fractures are among the most common reason for acute care hospitalization in individuals over age 65, with an estimated incidence of 340 000 fractures annually in the United States and over 1.5 million worldwide.^1,2^ While the benefit of operative management is widely accepted in terms of mobility, complication rates, long-term pain control and mortality,^3,4^ the optimal surgical timing is less well-defined. Data on surgical timing for hip fractures are limited by most analyses being retrospective and heterogeneous in terms of the study population and selected outcome measures, most specifically age criteria and time to the operating room (TTOR) goals, with cutoffs varying from 6-72 hours.^5-13^ Within these limitations, some have shown reduced 30-day mortality,^14^ reduced 1-year mortality,^15,16^ and lower complication rates^13^ for patients that undergo surgery less than 2 days from the time of admission.

Meta-analyses and population studies have been performed to identify the impact of early surgery more clearly. One population study showed a reduction in postoperative complications and 30-day mortality with TTOR 24 hours or less.^6^ Two independent meta-analyses have demonstrated reduced mortality,^9,11^ perioperative complications,^9^ and decubitus ulcer formation^11^ when the TTOR is less than 2 days.

The American Academy of Orthopedic Surgery (AAOS) published a performance measure in 2018 following a review concluding, ‘moderate evidence supports that hip fracture surgery within 48 hours of admission is associated with better outcomes.’^17^ The Association of Anaesthetists of Great Britain and Ireland made similar TTOR guideline recommendations.^18^ In line with available guidelines and evidence for a mortality benefit, our institution implemented a geriatric medicine/orthopedic surgery co-management pathway for individuals over age 65 with hip fractures. The pathway includes a goal TTOR of <24 hours, preoperative engagement with the geriatric medicine consult service, and multimodal pain management.

We sought to better understand 2 aspects of early surgical intervention that have not yet been fully addressed by existing literature and guidelines. Firstly, we aimed to evaluate the feasibility of early operative intervention. While many retrospective cohort studies have dichotomized TTOR as ‘early’ and ‘late,’ there is no pre-specified goal for TTOR. As a result, due to institutional, regional and national variation, the TTOR varies widely between studies. For example, in studies looking at TTOR <24 hours, the percentage receiving surgery within this time ranges from 24-55%^6,19,20^ In those looking at TTOR <48-hours, percentages range from 31.2-84.6%.^14,16,21^ Secondly, we quantified opioid pain medication use in the acute care setting, and the impact of surgical timing on oral morphine equivalent (OME) exposure. Opioids are commonly employed in this setting, yet most studies do not measure total opioid use in the pre- and post-operative setting of geriatric hip fractures.

The primary outcomes of this study were: to measure the impact of early (≤24 hr) vs delayed (>24 hr) TTOR on 1) hospital length of stay and 2) total and post-operative opiate use. Additional outcomes were: 1) the feasibility of reaching a prespecified target TTOR <24 hrs and 2) to identify potentially preventable etiologies associated with delayed TTOR.

## Materials and Methods

This study was an Institutional Review Board study approved by the University of California San Francisco. A retrospective review was performed of patients at a single tertiary referral center undergoing operative treatment for a hip or proximal femur fracture from January 2019 through January 2020.

**Figure 1.**
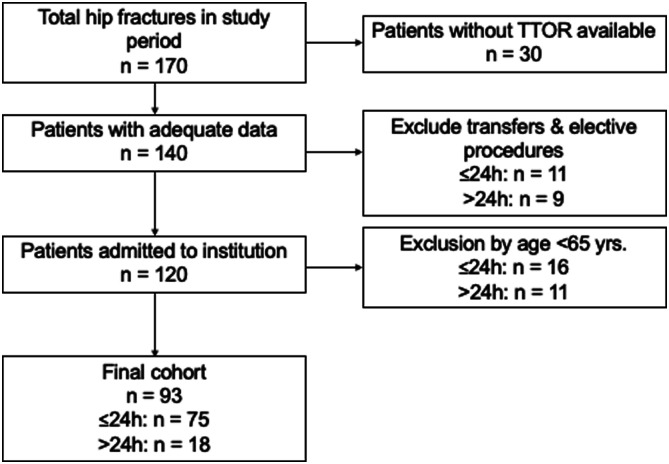
Patient selection flowchart.

The cohort of all hip fracture patients who underwent surgery during the study period was identified using an institutional database managed by the Division of Geriatrics. Patients were included if they were age 65 years or greater at the time of admission. Fractures of the hip and femur were included that met International Classification of Diseases (ICD)-10 diagnosis codes for fractures of these areas. Periprosthetic fractures and pathologic fractures meeting these criteria were also included. Patients with hip or femur fracture who were transferred to our institution from another acute care facility were excluded. Patients undergoing treatment for nonunion of prior fractures or elective surgical management were also excluded (Figure 1).

Initial data collected included patient height and weight, date of birth, and admission date and time. Opioid use was defined with oral morphine equivalents (OME). OME use was calculated preoperatively, in the recovery room, and throughout the hospitalization.

Patients’ primary speaking/reading language and use of surrogate decision maker consent for surgery were recorded. Furthermore, patients were categorized by whether they had a medicine consult, a geriatrics consult, or if they were admitted to an internal medicine care team primary service.

Two PGY5 orthopaedic residents (SA, ER) reviewed preoperative radiographs to classify fracture types by the Orthopaedic Trauma Association (OTA) classification^22^ and reviewed postoperative radiographs to denote implant types. If patients underwent advanced imaging for their fracture, the type of imaging, whether computed tomography (CT) or magnetic resonance imaging (MRI) was noted.

Admission time was classified into categories of weekend (Friday 6pm-Monday 6am) vs weekday (Monday 6am-Friday 6pm) and nighttime (6pm-6am) vs daytime (6am-6pm). Length of stay was calculated as the time between first recorded emergency department admission documentation and discharge time as recorded in the electronic medical record. Patient age was calculated based on the date of admission. Body mass index (BMI) was calculated from the hospital height and weight recorded in the electronic medical record.

The TTOR was calculated and stratified into 2 groups: ≤24.0 hours and >24.0 hours. Continuous variables were analyzed using 2 independent sample t-tests. Fisher’s Exact test was utilized to compare categorical variables. All statistical analyses were performed utilizing STATA v16.1 (StataCorp; College Station, TX). Significance was set at two-tailed *P*-value < .05.

## Results

### A Total of 93 Patients Were Included (Figure 1)

Baseline characteristics of the cohort are noted in Table 1. Mean age of the total cohort was 81.7 ± 8.8 years (range, 65.4-101.1 years). There was no difference between the ≤24 hour and >24-hour groups in age (*P* = .90) or BMI (*P* = .76). Pre-operative opioid use was not different between groups (*P* = .77). There were no differences in TTOR based on the admission day of the week (*P* = .63) or weekday vs weekend (*P* = .79). The >24-hour group had a higher proportion of patients arriving during daytime hours (88.9% vs 50.7%, *P* = .003). No differences were identified between groups on whether language spoken was English or the use of a surrogate decision maker.

**Table 1.** Baseline characteristics of the groups. Continuous variables are reported as mean ± standard deviation (range). Categorical variables are reported as n (%). Boldface *P*-values denote statistical significance (*P* < .05). TTOR: time to operating room. BMI: body mass index.

	TTOR < 24 hr (n = 75)	TTOR > 24 hr (n = 18)	*P*-value
Age (years)	81.8 ± 8.7	81.5 ± 9.7	.90
BMI (kg/cm^2^)	24.0 ± 4.3	23.6 ± 4.7	.76
Admission source			.77
Emergency room	23 (30.7)	4 (22.2)	
Skilled nursing facility	5 (6.7)	1 (5.6)	
Home	45 (60.0)	12 (66.7)	
Physician/Clinic Referral	2 (2.7)	1 (5.6)	
Admission time			.003
Daytime	38 (50.7)	16 (88.9)	
Nighttime	37 (49.3)	2 (11.1)	
Admission day			.79
Weekday	46	12	
Weekend	29	6	
Primary language			.27
English	46 (61.3)	14 (77.8)	
Other	29 (38.7)	4 (22.2)	
Surrogate decision-maker			.77
Yes	18 (24.0)	5 (27.8)	
No	57 (76.0)	13 (72.2)	
Preoperative opiate use			.77
Yes	18 (24.0)	5 (27.8)	
No	57 (76.0)	13 (72.2)	
Advanced imaging obtained for fracture evaluation			1.0
Yes	8 (10.7)	2 (11.1)	
No	67 (89.3)	16 (88.9)	

There were no differences in proportions of fracture types, type of surgery/implant, or use of spinal anesthesia type between groups (Table 2).

**Table 2.** Perioperative characteristics. Categorical variables are reported as n (%). TTOR: time to operating room. OTA: Orthopaedic Trauma Association. CRPP: closed reduction, percutaneous pinning. DHS: dynamic hip screw. IMN: intramedullary nail. ORIF: open reduction internal fixation. THA: total hip arthroplasty. ED: emergency department.

	TTOR ≤ 24 hr (n = 75)	TTOR > 24 hr (n = 18)	*P*-value
OTA fracture classification^22^			.70
31A1.2	9	1	
31A1.3	9	1	
31A2.2	6	1	
31A2.3	2	2	
31A3.1	3	1	
31A3.3	2	2	
31B1.1	5	1	
31B2.1	34	9	
31B2.2	1	0	
32A2b	3	0	
32A3b	1	0	
Surgical fixation/treatment			.51
CRPP	4 (5.3)	1 (5.6)	
DHS	1 (1.3)	0 (0)	
Hemiarthroplasty	24 (32.0)	7 (38.9)	
IMN	35 (46.7)	5 (27.8)	
ORIF	1 (1.3)	0 (0)	
THA/Revision arthroplasty	10 (13.3)	5 (27.8)	
Fascia iliaca block in ED			.39
Yes	20 (26.7)	7 (38.9)	
No	55 (73.3)	11 (61.1)	
Use of spinal anesthetic			1.0
Yes	15 (20.0)	3 (16.7)	
No	60 (80.0)	15 (83.3)	

Between the ≤24- and >24-hour groups, the time to OR significantly differed (17.4 ± 5.0 hours vs 33.1 ± 11.7 hours, respectively) (*P* < .00001) (Table 3). The total length of stay of the hospitalization, however, did not differ between groups, although there was a trend toward shorter length of stay in the ≤24-hr group (108.0 ± 67.2 hours for the ≤24-hr group vs 144.8 ± 103.7 hours for the >24-hour group, *P* = .066). There was no difference in post-op length of stay (90.6 ± 67.1 hours vs 111.7 ± 94.5 hours, *P* = .27).

**Table 3.** Time to OR, length of stay, and opioid use. Continuous variables are reported as mean ± standard deviation with range, min-max. Boldface *P*-values denote statistical significance (*P* < .05). TTOR: time to operating room. PACU: post-anesthesia recovery care unit. OME: oral morphine equivalents.

	TTOR ≤ 24 hr (n = 75)	TTOR > 24 hr (n = 18)	*P*-value
TTOR (hours)	17.4 ± 5.0 Range, 5-24	33.1 ± 11.7 Range, 25-70	< .00001
Total length of stay (hours)	108.0 ± 67.2 Range, 38-394	144.8 ± 103.7 Range, 48-506	.066
Post-operative length of stay (hours)	90.6 ± 67.1 Range, 25-389	111.7 ± 94.5 Range, 21-436	.27
Total OME	92.5 ± 188.0 Range, 0-1145	230.2 ± 296.7 Range, 0-847.5	.015
PACU OME	10.7 ± 23.0 Range, 0-146	16.2 ± 37.2 Range, 0-152.5	.43
Cumulative post-operative OME	81.3 ± 174.9 Range, 0-999	213.3 ± 271.3 Range, 0-765	.012
Average OME per hospital day	21.4 ± 48.1 Range, 0-381.7	42.5 ± 67.3 Range, 0-211.9	.13
Average OME per post-operative hospital day	26.3 ± 63.2 Range, 0-499.5	48.7 ± 78.4 Range, 0-255	.20

Total opioid use throughout the hospitalization significantly differed between groups (92.5 ± 188.0 OMEs for the ≤24-hr group vs 230.2 ± 296.7 OMEs for the >24-hour group, *P* = .015). OME use in the post-anesthesia recovery unit did not differ (*P* = .43). Cumulative post-operative opioid use significantly differed (81.3 ± 174.9 OMEs for the ≤24-hr group vs 213.3 ± 271.3 OMEs for the >24-hour group, *P* = .012). There were no significant differences when analyzing OME use by average per hospital day (*P* = .13) and average per postoperative hospital days (*P* = .20) (Table 3).

## Discussion

Hip fracture surgery within 24 hours of hospitalization for individuals over 65 was achievable with >80% of cases performed within this time frame. Furthermore, early surgery was associated with a significant reduction in total and postoperative opiate use in our study population. These findings align with prior studies demonstrating that early surgery is associated with reduced pain in older adults.^23^ Interestingly, none of the other patient specific factors that we evaluated (fracture type, use of surrogate decision maker, primary language, preoperative imaging, etc.) were different between our study groups, suggesting that surgery <24 hours from admission is a key determinant. This contributes to the growing body of literature supporting prompt surgical treatment to reduce overall morbidity after hip fracture.^9,11,15-17^

Multimodal perioperative pain management is an especially important aspect of hip fracture care, as post-surgical pain can delay early mobilization and rehabilitation.^24,25^ Efforts to reduce peri and post-surgical opiate use are critical, as older adults are at higher risk for opiate-related side-effects such as constipation, delirium, falls, and medication interactions. Higher doses of opiate medications also increases the risk of developing long-term opioid dependence and addiction, in addition to other side effects of constipation, respiratory depression, delirium, and interactions with other medications, to name a few.^25-28^ Multimodal pain management is an important pillar of hip fracture post-surgical care, as it has been shown to improve the efficacy of pain management and reduce opiate use.^24,29^ In the present study, patients admitted for hip fracture surgery were all treated with a multi-modal pain regimen created through a collaboration with the anesthesia/pain management service, emergency medicine, orthopedic surgery and geriatric medicine. Nearly half of the patients received fascia iliaca blocks in the emergency department, an intervention which has shown to decrease inpatient opiate requirements in hip fracture patients.^30-32^ In addition to specific pain management strategies, it appears that implementing a goal of <24 hours from admission to hip fracture surgery may present an opportunity to lessen opiate use and improve post-surgical care.

Although the total and cumulative post-operative OME use was significantly different between our study groups, the average OME use per hospital day was not different. In aggregate, this data suggests that faster time to surgery may lead to sooner hospital discharge, thereby reducing total opiate use. While it would be useful to assess the long-term impact of early surgery on patterns of pain medication use, this study did not look at opiate use post-discharge and thus we were not able to assess opiate consumption trends between the 2 groups in the outpatient setting. Future studies capturing patient-reported pain scores and functional scales would help in understanding the impact of early surgery on the patient’s experience and functional recovery.

This study also demonstrated that surgical management of geriatric hip fractures within 24 hours of admission is feasible, with 81% of the present cohort undergoing surgery in 24 hours or less. This is meaningfully higher than the percentages reported in several other studies from other institutions (range 24-55%)^6,19,20^ and may be attributed in large part to the implementation of a multidisciplinary hip fracture pathway at our institution.^33^ This pathway was designed to standardize and optimize care for patients ≥65 years old with hip fracture. A recently published study at our institution with a different timeframe and slightly different inclusion criteria noted an increase from baseline proportion of surgeries for hip fragility fractures within 24 hours from 55.9% to 78.6% (*P* = .037).^34^ Key aspects included: a goal time to OR of 24 hours or less, clearly defined guidelines for preoperative medical optimization, pain control and anticoagulation management, as well as scheduling operating room time in advance for an anticipated hip fracture surgery, which likely helped to expedite care and avoid delays for many of our patients. Individuals <65 years-old over the same time frame at our institution were not treated under the geriatrics orthopedic surgery co-management protocol and were less likely to go to surgery in 24 hours or less (59% vs 81%).

As the prevalence of hip fracture continues to rise in our aging population, the economic impact of prolonged hospitalizations will need to be considered. In this study, hip fracture repair surgery performed more than 24 hours after admission was not associated with increased hospital length of stay, although there was a trend toward this direction. This contrasts with previous studies that have demonstrated that early surgery is associated with reduced length of stay.^16,35-38^ In the present cohort, this discrepancy may have stemmed from a substantial portion of the >24 hour group receiving surgery in a time window that was over 24 hours, but did not increase the number of hospital days (ie TTOR between 25-30 hrs), or inadequate sample size.

Identifying modifiable factors leading to surgical delay is important to focus interventions aimed at reducing surgical delays. In our cohort, we considered patient-related factors, process factors, and team factors to see if any were significantly different in the >24 hour group yet found no difference in most of the potential factors for delay (advanced imaging at time of admission, limited English proficiency, need for surrogate decision maker, and weekend admission). However, we did find that daytime admission (ED encounter beginning between 6a-6p) was significantly associated with TTOR >24 hours. With the goal of medical optimization (including medical consultation and/or testing) prior to surgery and having adequate operating time and staff, it may be less feasible for an afternoon admission to undergo surgery within 24 hours, which may contribute to the observed delay.

This study has several limitations. While retrospective chart review is adequate for determining TTOR, OME administration, LOS, and surgical details, it is limited in the ability to identify causes for surgical delay, or to quantify the contribution of specific factors in delaying TTOR. Documentation is often incomplete in this area, and it is often not feasible to make accurate inferences about causes for delay. Additionally, the sample size in the present cohort is small, particularly in the delay group. While this supports our institution’s goal is to limit the number of patients with delayed care, larger scale and multi-institutional studies may provide more robust data for comparison. Prospective studies would provide an opportunity to determine causes for delayed operative treatment and may identify areas for quality improvement to reduce modifiable risks for delay. We were unable to effectively capture patient medical complexity and frailty, which may further contribute to differences in surgical timing and opiate consumption.

## Conclusions

Surgical treatment of geriatric hip fractures in 24 hours or less from the time of ED presentation is achievable and was associated with reduced inpatient opiate use. Establishing institutional TTOR goals as part of an interdisciplinary hip fracture co-management clinical pathway can facilitate prompt care and contribute to recovery in these patients with highly morbid injuries.
